# An unusual presentation of fungal brain abscess in immunocompetent host

**DOI:** 10.12669/pjms.40.6.8877

**Published:** 2024-07

**Authors:** Sajid Islam Bhatti, Sidra German, Tajammul Waqar, Rubina Naqvi

**Affiliations:** 1 Sajid Islam Bhatti Department of Nephrology, Sindh Institute of Urology and Transplantation (SIUT), Karachi. Pakistan; 2 Sidra German Department of Nephrology, Sindh Institute of Urology and Transplantation (SIUT), Karachi. Pakistan; 3 Tajammul Waqar Department of Nephrology, Sindh Institute of Urology and Transplantation (SIUT), Karachi. Pakistan; 4 Rubina Naqvi Department of Nephrology, Sindh Institute of Urology and Transplantation (SIUT), Karachi. Pakistan

**Keywords:** Kidney Replacement Therapy (KRT), Acute Kidney Injury (AKI)

## Abstract

Focal area of necrosis, with a surrounding membrane within the brain parenchyma, usually resulting from an infectious process or rarely from a traumatic process known as brain abscess. We report a case of young female, who presented with multiple abscess in left frontal and right occipital region of brain, she was otherwise immunocompetent, lacking any known risk factor for opportunistic infection. And this fungal abscess manifest with unusual presentation of bilateral lower limb weakness along with seizures and fever. This infection leads to acute kidney injury (AKI), necessitating kidney replacement therapy (RRT) in term of intermittent hemodialysis (IHD). After drainage of abscess and antifungal therapy, she responded well, her acute kidney injury resolved and she showed clinical and radiological improvement.

## INTRODUCTION

Focal area of necrosis, with a surrounding membrane within the brain parenchyma, usually resulting from an infectious process or rarely from a traumatic process, known as brain abscess.^1^ It may be originated from infections in surrounding area of head and neck or spread from distant organs as hematogenous seedling, known as primary brain abscess. Those that occur after brain injury or brain surgery, these abscesses are labelled secondary brain abscess.^2^ It could be bacterial, viral, fungal or parasitic (protozoal or helminthic). The incidence has been estimated to range from 0.2 to 1.9 per 100 000 person-years, incidence has been increased in those who are above 40 years of age and those who are immunocompromised.^3^ Fungal abscesses are more prevalent in state of decreased immunity like AIDS, cancers, patients with solid organ or hematological transplantation, diabetes mellitus, patients getting chemotherapy, individuals with IV drug abuser, state of neutropenia and hereditary immune defects.^4^

We report a case of young female, who presented with multiple abscess in left frontal and right occipital region of brain, she was otherwise immunocompetent, lacking any known risk factor for opportunistic infection.

## CASE PRESENTATION

A 30-year-old female, married, resident of Sindh, with no known prior co-morbid, gave birth to her first child at 35 weeks of gestation on 20^th^ June 2023. Her antenatal period was unremarkable except for raised pressures in last week prior to delivery. There was no history of increase blood pressures, abnormally raised blood sugars, body swelling, antepartum or postpartum hemorrhage or any other autoimmune history. Due to raised blood pressure her obstetrician induced labor at 35 weeks of gestation and child was delivered successfully via normal vaginal delivery aided with episiotomy at a private setting clinic/hospital. She was discharged to home same day of child birth. After 24 hours of discharge from hospital she developed raised temperature which recorded up to 101^0^F. Patient went to same hospital found to have retained products of conception. She underwent evacuation of uterine cavity and repair of 3^rd^ degree perineal tear. Despite of antibiotics, she remained febrile, later oligo-anuric, developed AKI necessitates kidney replacement therapy (KRT) in term of hemodialysis. During hospital stay, she developed generalized tonic-colonic seizures, therefore brain imaging (CT scan) performed which was reported normal.

Presented to this hospital on 1^st^ of July 2023 with complaints of fever for one week, anuria, generalized seizures, bilateral lower limb weakness for three days and altered level of consciousness for one day. Apart from current illness her past medical and surgical history was insignificant and family history was also unremarkable. On arrival her vitals were BP 110/66, pulse 110 beats /minutes, Respiratory rate 20 breaths/ minutes, Temperature 99^0^F and blood sugar was 120mg/dl. She was pale and had mild pitting edema on both shins, however, there was no cyanosis, clubbing, jaundice, koilonychia or raised JVP. Her neurological examination revealed Glasgow Coma Scale (GCS) of 12/15 (E = 4, V= 4, M= 4), neck supple, pupil bilaterally equal and reactive to light, tone was reduced in all four limbs, power 3/5 in lower limbs and 4/5 in upper limbs, reflexes were diminished and planters withdrawal bilaterally. Chest, cardiovascular and abdominal examination was unremarkable, area of episiotomy was clean.

On presentation her initial labs were hemoglobin 8.9 g/dl, TLC 9.5x10^9^/ L, Platelet 70,000/L. Urea 213mg/dl, Creatinine 8.3mg/dl, Sodium 145meq/L, K 3.9meq/L, Chloride 100meq/L, Bicarbonate 7meq/L, Calcium 7.0mg/dl, phosphorus 9.9mg/dl, Albumin 2.3mg/dl. Total bilirubin 0.75mg, Direct bilirubin 0.18mg %, Alkaline phosphatase 78U/L, SGOT 73U/L, SGPT 171U/L while GGT was 62U/L. prothrombin time (PT) 11.4 sec, APTT 20.8 sec and INR 1.08. Urine D/R showed protein 4+, RBCs 5+ and while pus cells +++ (Table-I). On ultrasound both kidneys were of normal size and texture, chest roentgenogram and electrocardiography were normal. For her confused mentation and weakness of limbs first magnetic resonance imaging (MRI) of brain with contrast done on 3^rd^ July 2023 which showed multiple rings enhancing lesions in different regions of brain (Fig.1).

**Table-I:** Laboratory investigations.

work-up	01-07-2023	15-07-2023	21-07-2023	14-09-2023
Serum Creatinine	8.33 mg/dl	4.95 mg/dl	3.4 mg/dl	0.29 mg/dl
Blood urea	213 mg/dl	198 mg/dl	144 mg/dl	35 mg/dl
Serum Sodium	145mEq/L	142 mEq/L	140 mEq/L	138 mEq/L
Serum Potassium	3.9 mEq/L	4.6mEq/L	3.9 mEq/L	3.8 mEq/L
Serum Chloride	100 mEq/L	98 mEq/L	98 mEq/L	106 mEq/L
Serum HCO^3^	17mEq/L		26 mE/l	16 mEq/L
ALT	171 U/L			113 U/L
AST	73 U/L			147 U/L
Alkaline Phosphatase	78 U/L	71U/L	80 U/L	553 U/L
GGT	62 U/L			741 U/L
Total bilirubin	0.75 mg/dl			1.11 mg/dl
Direct bilirubin	0.18 mg/dl			0.46 mg/dl
LDH	784 U/L			
Total protein	5 g/dl			
Serum globulin	2.7g/dl			
Serum albumin	2.3 g/dl	2.4 g/dl	2.5 G/dl	
A/G ration	0.9			
Serum Calcium	7.01 mg/dl	7.73 mg/dl	8.56 mg/dl	
Serum Phosphorus	9.9 mg/dl	4.0 mg/dl	5.8 mg/dl	
HbsAg	Non-reactive			
Anti-HCV	Non-reactive			
HIV	Negative			

	*01-07-2023*	*15-07-2023*	*21-07-2023*	*14-09-2023*

Hb g/dl	8.9	8.4	9.9	10.3
Hct %	28.2	26.5	30.5	30.8
MCV	82.1	88.9	82.7	87
WCC	9.57	11.3	16.66	16.7
Platelets	70	117	218	299
PT	11.4			
APTT	20.8			
INR	1.08			

**MRI BRAIN (Fig1):**
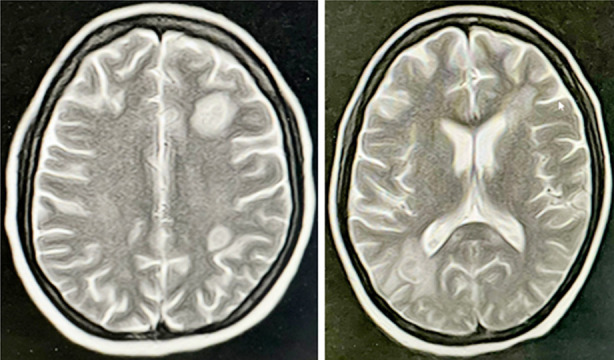
Multiple well defined abnormal signal intensity areas are seen in left frontoparietal region, left paraventricular, right occipital region and right cerebellar hemisphere with adjacent perilesional edema. They are showing peripheral ring enhancement. These lesions are characteristic of abscesses.

Cerebral spinal fluid analysis was done which showed mildly raised protein of 90mg/dl while total leucocyte count was nil. As her MRI did not explain weakness and CSF was showing albumin-cytologic dissociation so nerve conduction study was planned. NCVs showed acute motor axonal polyneuropathy. This finding was in favor of Guillain Bare Syndrome (GBS). Neurology team was taken on board and they advised to initiate plasmapheresis and a total of six session of plasmapheresis were done. Meanwhile patient was treated with Carbapenem, while Colistin was added later on to, as she continues to exhibit spiking fever, she was also given intermittent vancomycin and phenytoin. MRI was discussed with neurosurgeon and they advised to empirically add intravenous Amphotericin, therefore, prior adding Amphotericin, toxoplasmosis serology and serum galactomannan levels were sent on 12^th^ July 2023. Patient deteriorated further and did not respond to plasmapheresis, weakness progressed and had respiratory paralysis, so she was intubated on 14^th^ July 2023 and kept on mechanical ventilatory support.

On 14^th^ July CT brain repeated which showed further enhancing of ring lesions and cerebral edema was progressing further (Fig.2). Again, neurosurgery consult was obtained for the drainage of the abscess. Left frontal abscess was drained after bur hole procedure and samples for gram stain, microbial culture, AFB smear, fungal smear and fungal culture were sent. Her all cultures and AFB was negative except fungal smear which was positive on KOH stain and it showed fungal hyphae but culture did not show growth. Meanwhile toxoplasmosis serology also turned out to be positive but galactomannan level was found negative. Trimethoprim/Sulphamethoxazole added to treatment regimen for a duration of four weeks. Her urine output and renal functions started improving and she became dialysis independent, her last dialysis was performed on 19^th^ July 2023.

**CT SCAN BRAIN (FIG 2):**
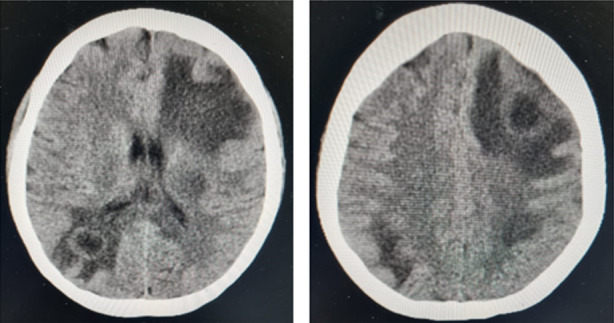
Multiple well defined peripherally enhancing hypodense area in left fronto-parietal region, left paraventricular, right occipital and right cerebellar hemisphere with adjacent perilesional edema as suggestive of abscess formation.

As serum creatinine became normal, she was switched to liposomal Amphotericin and tracheostomy was made on 2^nd^ august 2023. Gradually she started improving her GCS and tracheostomy was closed on 14^th^ august 2023, she was switched to oral voriconazole, serum creatinine remained less than 1mg/dl. MRI repeated on 4^th^ sept 2023 and it showed lesions in healing phase (Fig.3). Patient responded well, both clinically and radiologically. She was discharged to home on 14^th^ September on voriconazole 200mg bid and levetiracetam, former has to be continued for six months duration. Phenytoin was switched to levetiracetam due to drug-drug interaction between voriconazole and phenytoin, as phenytoin decreases therapeutic levels of voriconazole. She in on regular follow-up as an out-patient.

**MRI BRAIN (FIG 3):**
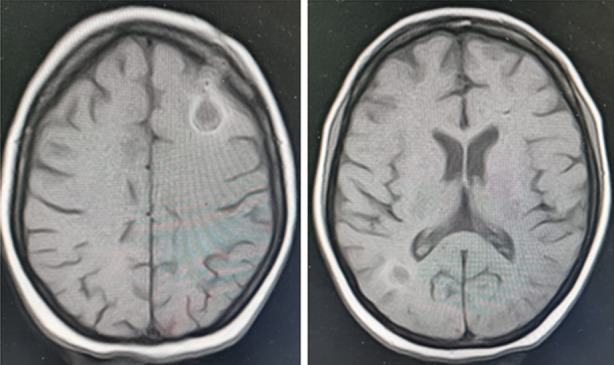
after burr hole it showed lesion in left fronto-parietal and right occipital region, reduced in intensity and in healing phase.

## DISCUSSION

Any trauma or infectious process that leads to localized area of necrosis with surrounding membrane in brain parenchyma is known as brain abscess. Its incidence is about 8 % in developing countries, and 1 - 2 % in developed countries.^1^ It is potentially life threatening and can lead to persistent neurological sequelae.^5^ It is two to three times more prevalent in men and morbidity rate is highest in fourth decade of life.^6^ Its most common presentation include headache, seizures, altered level of consciousness, vomiting and vision changes.^7^ Usually manifestation seems to be nonspecific, therefore cause delay in diagnosis. It is usually due to site and size of the lesions.

Direct or indirect invasion from surrounding structures like teeth, paranasal sinuses and ear remains the most common source of microbial infection.^8^ Traumatic or penetrating brain injury can also be the source of microbial entry. However, regardless of all these sources, in 20 - 30 % of cases source remains unknown and it is known as cryptic brain abscess.^8^ Diagnosis relies on cerebrospinal fluid analysis, along with brain imaging computed tomography and magnetic resonance imaging. It shows well defined ring enhancing lesion and features of cerebral edema in stage of cerebritis. Definitive diagnosis requires microbiological examination of abscess for gram stain, microbial culture, fungal smear and culture and stain for acid fast bacilli and mycobacterium culture.^9^ Treatment requires aspiration and excision of abscess followed by antibacterial, antifungal or anti-tuberculous treatment as per cultures result.^9^

Our case in unusual because she is young female with no prior morbidities, she lack any factor for immunocompromised state and yet develops fungal abscess with unusual presentation of limb weakness along with seizures and fever. She had only significant history of gynecological procedure probably from there she acquired fungal spores from environment or instruments used during procedure and this lead to this extensive fungaemia and toxoplasmosis. Fungal abscess are usually seen in immunocompromised state like diabetes, AIDS, malignancy, chemotherapy and those who have got solid organ or hematological transplantation. Usually, fungal aspergillus species has been seen in transplant recipients but unusual opportunistic molds can also be identified in some cases. 10 Our patient responded to antifungal voriconazole and showed clinical and radiological improvement and she is at regular follow up as outpatient.

## CONCLUSION

Even in immunocompetent state fungal abscess should be kept in differential diagnosis and sample should be sent for fungal smear and cultures as well. Unusual presentation should not mislead from diagnosis and pan cultures should be requested and source should be identified.

### Authors’ Contribution:

**SB:** Prepared manuscript and guided through sequencing the diagnostics and literature work.

**SG:** Identified the case, thought about presenting it.

**TW:** Assisted through patient going under all follow-up and investigation and literature search.

**RN:** Significantly revised the manuscript.
